# Biomarkers Associated with Failure of Liberation from Oxygen Therapy in Severe COVID-19: A Pilot Study

**DOI:** 10.3390/jpm11100974

**Published:** 2021-09-29

**Authors:** Oh Joo Kweon, Min Jae Cha, Moon Seong Baek, Seong-Ho Choi, Won-Young Kim

**Affiliations:** 1 Department of Laboratory Medicine, Chung-Ang University Hospital, Chung-Ang University College of Medicine, Seoul 06973, Korea; kwonpoi99@caumc.or.kr; 2 Department of Radiology, Chung-Ang University Hospital, Chung-Ang University College of Medicine, Seoul 06973, Korea; minjaecha@cau.ac.kr; 3 Department of Internal Medicine, Division of Pulmonary and Critical Care Medicine, Chung-Ang University Hospital, Chung-Ang University College of Medicine, Seoul 06973, Korea; wido21@cau.ac.kr; 4 Department of Internal Medicine, Division of Infectious Diseases, Chung-Ang University Hospital, Chung-Ang University College of Medicine, Seoul 06973, Korea; tobeservant@cau.ac.kr

**Keywords:** angiopoietins, biomarkers, COVID-19, endocan, SARS-CoV-2, weaning

## Abstract

This study aimed to investigate whether clinical and laboratory biomarkers can identify patients with COVID-19 who are less likely to be liberated from oxygen therapy. This was a retrospective study comparing 18 patients in the weaning failure group with 38 patients in the weaning success group. Weaning failure was defined as death or discharge with an oxygen device before day 28 after hospital admission or requiring oxygen support as of day 28. The median quick Sequential Organ Failure Assessment (qSOFA) score was significantly higher and the median SpO_2_/FiO_2_ was significantly lower in the weaning failure group. The laboratory biomarkers, procalcitonin (PCT) and D-dimer, were significantly higher in the weaning failure group, as were the biomarkers of endothelial injury, such as angiopoietin-2 (Ang-2) and Ang-2/Ang-1, and tumor necrosis factor-α (TNF-α). Patients’ qSOFA scores, SpO_2_/FiO_2_, and PCT, D-dimer, Ang-2, Ang-2/Ang-1, endocan (4-day and 7-day increases), and TNF-α levels predicted weaning failure; 7-day endocan levels were the best predictor of weaning failure with an AUC of 0.81 (95% CI, 0.67–0.94). We identified clinical and laboratory parameters, including plasma biomarkers of endothelial injury, that may be considered as biomarkers for predicting failure of liberation from oxygen therapy in patients with severe COVID-19.

## 1. Introduction

Coronavirus disease 2019 (COVID-19) has now spread to over 220 countries worldwide, approaching 224 million cumulative cases and reaching 4,627,540 deaths as of 14 September 2021 [1]. Although most patients develop a mild form of COVID-19, 5–10% may progress to more severe forms of the disease, often requiring intensive care [2]. Activation of inflammatory cascades, endothelialitis, capillary leakage resulting in acute respiratory distress syndrome (ARDS), thromboembolism, and multiorgan failure are observed among patients with severe COVID-19 [3].

Early identification of laboratory predictors of progression to severe COVID-19 has been extensively studied, as valid predictors may help guide risk stratification and clinical management, allocate limited medical resources, and target patients for interventional studies. Recent meta-analyses of numerous studies worldwide have found significant associations between hematologic, biochemical, coagulation, and inflammatory biomarkers and COVID-19 severity [4,5]. In addition, serum endothelial/epithelial molecules, such as endocan and soluble receptor for advanced glycation end-products (sRAGE), were found to predict mortality and to indicate the need for intensive care [6,7].

Patients with severe COVID-19 may not be functionally independent at hospital discharge due to old age, comorbidities, and/or prolonged mechanical ventilation (MV). In a recent study investigating the functional status of COVID-19 survivors receiving MV, half of the patients were discharged with supplemental oxygen [8]. The early prediction of the functional status of these patients at the intensive care unit (ICU) or hospital discharge may allow for early rehabilitation and optimization of hospital bed management in the current pandemic. However, primary outcomes in previous biomarker studies were confined to initial severity of illness and mortality [4,5]. To date, no study has evaluated predictors associated with failure of liberation from oxygen therapy among patients with severe COVID-19.

This study aimed to analyze clinical and laboratory parameters in patients with severe COVID-19 to discriminate patients who were less likely to be liberated from oxygen therapy. Baseline values and changes during the 7-day study period were assessed to account for dynamic changes of biomarkers in terms of predicting outcomes.

## 2. Materials and Methods

### 2.1. Study Design

In this retrospective single-center study, we enrolled confirmed COVID-19 pneumonia cases in an 835-bed university-affiliated tertiary hospital in Seoul, Republic of Korea. Patients were hospitalized between June 2020 and March 2021. We excluded patients who did not receive oxygen or who had substantial missing data. For the present study, we measured plasma biomarkers of endothelial/epithelial injury and inflammation using prospectively collected blood samples for the investigation of clinical characteristics and viral shedding among patients with COVID-19. Written informed consent was obtained from the patient or their next of kin. The study was approved by the Chung-Ang University Hospital Institutional Review Board (2092-001-432) and was conducted in accordance with the principles of the Declaration of Helsinki.

The study patients were divided into two groups according to oxygen therapy status at day 28 after hospital admission. The weaning failure group comprised patients who died or required any form of oxygen support at day 28. Patients who were discharged to long-term care (at a medical center or at home) with MV or low-flow oxygen therapy before 28 days were included in the weaning failure group. The weaning success group comprised patients who were alive and required no oxygen therapy at day 28. Baseline data, clinical outcomes, and changes in laboratory parameters were compared between the two groups.

### 2.2. Data Collection

The confirmation of COVID-19 was based on the positive detection of the viral genome in upper respiratory tract specimens using the reverse transcription polymerase chain reaction (RT-PCR) assay [9]. Baseline data included demographics (age and sex), body mass index, comorbidities, the presence of ARDS [10], bacterial coinfection, patients’ quick Sequential Organ Failure Assessment (qSOFA) score [11], initial oxygen therapy, and prescribed medications. Vital signs, laboratory data, radiographic findings, RT-PCR results, and endothelial/epithelial biomarker values were collected initially and during follow-up. Laboratory data comprised a complete blood count and liver as well as renal function tests, in addition to electrolyte, lactate dehydrogenase (LDH), C-reactive protein (CRP), ferritin, procalcitonin (PCT), D-dimer, and serum glucose levels. The radiologic score was calculated by reviewing anteroposterior chest radiographs, as previously described [12]. The viral load is expressed as the cycle threshold (Ct) value, which is inversely correlated with the amount of viral RNA. We also collected biomarkers of endothelial injury (angiopoietin-1 [Ang-1], Ang-2, the soluble form of the Tie2 receptor, endocan, intercellular adhesion molecule-1, syndecan-1, and the von Willebrand factor), epithelial injury (sRAGE and surfactant protein D [SP-D]), and inflammation (interleukin-6 [IL-6] and tumor necrosis factor-α [TNF-α]).

### 2.3. Laboratory Testing

Simultaneous measurements of biomarkers of endothelial/epithelial injury and inflammation were conducted within plasma samples using the Luminex^®^ Assay Human Premixed Multi-Analyte Kit (R&D Systems, Minneapolis, MN, USA). The assay was performed according to the manufacturer’s instructions. In brief, 50 μL of the sample and microparticle solution were added to each well of the 96-well microplates. After incubation and washing, a biotin-antibody solution was added. Microplates were incubated and washed again; a streptavidin–phycoerythrin solution was added, followed by another washing procedure. After resuspending the microparticles of each well with a washing buffer, multiple signals generated from each well were read and converted to concentrations using the MAGPIX^®^ Multiplexing System (Luminex Corporation, Austin, TX, USA) with Bio-Plex Manager MP/Bio-Plex Manager 6.1 software (Bio-Rad Laboratories, Inc., Hercules, CA, USA). Measurements of biochemical parameters were performed using standard laboratory techniques.

### 2.4. Statistical Analyses

Continuous variables were reported as medians and interquartile ranges (IQRs), and categorical variables were reported as numbers and percentages. We compared differences in demographics, clinical variables, and laboratory parameters between the weaning failure and success groups through the Mann-Whitney *U* test (for continuous variables) and through the chi-squared test or Fischer’s exact test (for categorical variables). To assess dynamic changes in biomarker levels during the study period, the areas under the curve (AUCs) were calculated for clinical variables and laboratory parameters with repeated measurements (baseline, day 4, and day 7), as previously described [13]. The AUCs were compared between the weaning failure and success groups using the Mann-Whitney *U* test. Receiver operating characteristic curve analysis was used to determine the ability of different biomarkers to predict weaning failure. Identification of an optimal cut-off value for each variable of interest was based on Youden’s index [14]. Kaplan-Meier estimates were stratified according to the cut-off derived from Youden’s index to estimate weaning failure. All tests were two-sided, and *p*-values < 0.05 were considered statistically significant. Data analyses were performed using IBM SPSS software (version 26.0; IBM Corp., Armonk, NY, USA) and MedCalc for Windows (version 19.8; MedCalc Software, Ostend, Belgium).

## 3. Results

We initially screened 77 consecutive patients with COVID-19 pneumonia who were admitted to our hospital between June 2020 and March 2021. Twenty patients who did not receive oxygen therapy as well as a patient who had missing baseline laboratory data were excluded from the analysis. A total of 56 patients with severe COVID-19 were included in this study (18 patients in the weaning failure group and 38 patients in the weaning success group).

### 3.1. Patient Characteristics

Patients’ baseline characteristics and clinical outcomes are described in Table 1. Patients in the weaning failure group were significantly older than those in the weaning success group (median: 76 years [IQR: 68–79 years] vs. 68 years [IQR: 57–71 years]; *p* = 0.01). ARDS (50% vs. 21%; *p* = 0.03) and MV (72% vs. 24%; *p* = 0.001) were more prevalent in the weaning failure group. The median qSOFA score was also significantly higher in the weaning failure group (2 [IQR: 1–2] vs. 0 [IQR: 0–1]; *p* = 0.003), whereas the median pulse oximetric saturation to fraction of inspired oxygen ratio (SpO_2_/FiO_2_) was significantly lower (157 [IQR: 124–190] vs. 208 [159–297]; *p* = 0.004). Further, the median respiratory rate was significantly higher (25 breaths/min [IQR: 24–32 breaths/min] vs. 20 breaths/min [IQR: 20–26 breaths/min]; *p* = 0.006) in the weaning failure group. We did not detect a statistical difference in initial chest radiography or viral load between the groups.

Compared with the weaning success group, the weaning failure group had longer hospital stays, higher hospital mortality, and higher proportions of hospital-acquired infections (Table 1). In the weaning failure group, 39% (7/18) patients died, and 28% (5/18) patients needed some form of oxygen support at day 28; 22% (4/18) and 11% (2/18) of patients were discharged with supplemental oxygen or MV, respectively.

### 3.2. Laboratory Data

Routine laboratory data are shown in Table 1. The platelet count was significantly lower in the weaning failure group, and PCT and D-dimer levels were significantly higher. Table 2 shows endothelial/epithelial and inflammatory biomarker values. Ang-2 and Ang-2/Ang-1, which represent the degree of endothelial injury, were significantly higher in the weaning failure group. Moreover, patients in the weaning failure group had a significantly higher level of TNF-α (a biomarker of inflammation).

**Figure 1:**
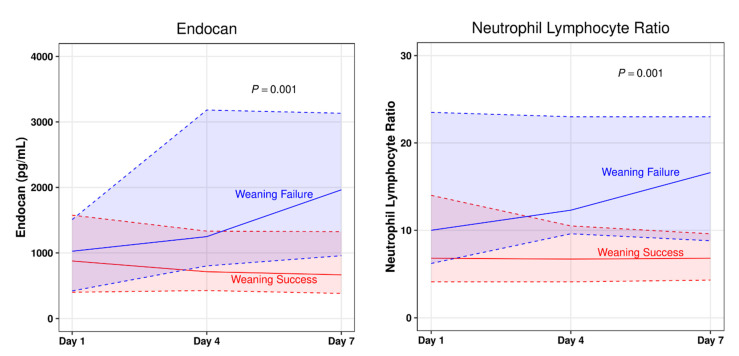
Plasma endocan levels and neutrophil lymphocyte ratios during the 7-day study period. Solid lines represent median values, and the shaded areas bordered by dotted lines represent upper and lower quartiles. The AUCs were compared between groups using the Mann-Whitney *U* test. AUC: area under the curve.

Seven-day changes in clinical variables and laboratory parameters were assessed to account for dynamic changes in biomarker levels in terms of predicting weaning failure. Only endocan (median AUC: 688 pg/mL [IQR: 235–1257 pg/mL] vs. −59 pg/mL [IQR: −318 to 174 pg/mL]; *p* = 0.001) and neutrophil lymphocyte ratio (NLR) (median AUC: 1.8 [IQR: 0.5–6.2] vs. −0.4 [IQR: −5.1 to 2.1]; *p* = 0.04) changes were significantly different between the groups (Figure 1). Serial analyses of the other biomarkers are described in Table S1.

### 3.3. Predictors of Weaning Failure

**Figure 2:**
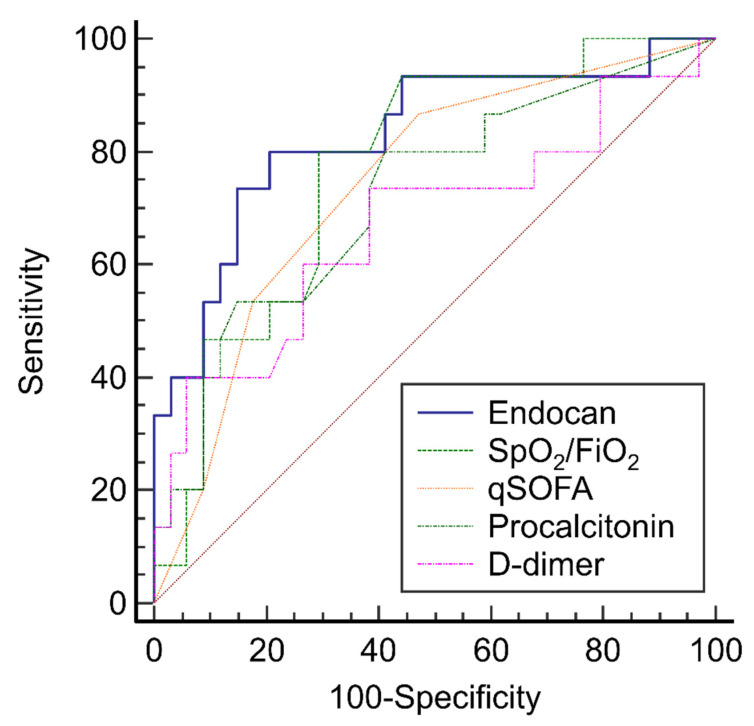
Comparison of ROC curves for predicting weaning failure. FiO_2_: fraction of inspired oxygen; SpO_2_: pulse oximetric saturation; qSOFA: quick Sequential Organ Failure Assessment; ROC: receiver operating characteristic curve.

**Figure 3:**
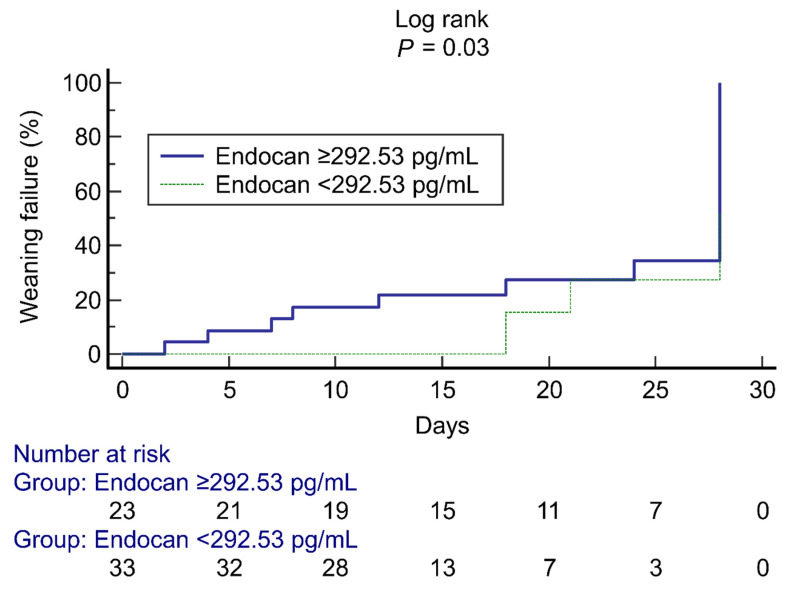
Kaplan-Meier curves showing the cumulative number of weaning failure (death or discharge with oxygen support) patients before day 28 after hospital admission based on a 7-day increase in endocan. The bottom of the figure shows the number of patients with severe COVID-19 admitted over time. COVID-19: coronavirus disease 2019.

When comparing clinical variables and laboratory parameters between the weaning failure and success groups, nine biomarkers were significantly different within the univariable analyses, including qSOFA score, SpO_2_/FiO_2_, PCT, D-dimer, Ang-2, Ang-2/Ang-1, endocan (4-day and 7-day increases), and TNF-α (Table 1 and Table 2). These nine biomarkers were selected to construct a prediction model for weaning failure (Table 3). The 7-day increase in endocan (AUC: 0.81, 95% confidence interval, 0.67–0.94; *p* < 0.001) was the best predictor when compared with SpO_2_/FiO_2_, SOFA score, PCT, and D-dimer (Figure 2). The optimal cut-off for the 7-day increase in endocan (to predict weaning failure) was 292.53 pg/mL based on Youden’s index, with a 78% sensitivity and 79% specificity. Cumulative weaning failure assessed by a Kaplan-Meier curve with a cut-off of 292.53 pg/mL demonstrated a distinct separation between the groups (Figure 3); 61% (14/23) of patients with a 7-day increase in endocan of ≥292.53 pg/mL failed to be liberated from oxygen therapy at day 28, whereas only 12% (4/33) of patients with endocan <292.53 pg/mL failed. Models for predicting weaning failure through other biomarkers are described in Table S2.

## 4. Discussion

Although numerous studies have examined COVID-19 variables associated with disease severity and mortality, few studies have evaluated the functional status of COVID-19 survivors, such as with respect to oxygen therapy at ICU or hospital discharge. This study investigated predictors associated with failure of liberation from oxygen therapy at day 28 after hospital admission among patients with severe COVID-19. For baseline variables, qSOFA score, SpO_2_/FiO_2_, PCT, D-dimer, Ang-2, Ang-2/Ang-1, and TNF-α were found to be the moderate predictors of weaning failure. Notably, the dynamic change in endocan during the 7-day period was the best performing biomarker in terms of predicting weaning failure.

In the present study, baseline qSOFA score and SpO_2_/FiO_2_ significantly predicted weaning failure. These findings are not surprising, because patients who are initially tachypneic with low oxygen saturation are more likely to be mechanically ventilated. Patients with a longer MV duration receive sedatives and neuromuscular blockers for longer periods of time [15]. Among patients with COVID-19 ARDS, continuous neuromuscular blockers were prescribed to approximately 84% of patients [16], whereas all of the 22 patients undergoing MV in our study received neuromuscular blockers. It is possible that patients with severe COVID-19 are at risk of prolonged bed rest, leading to muscle weakness and decreased pulmonary function, thus decreasing the likelihood of liberation from oxygen therapy.

PCT is often implemented as a biomarker of systemic bacterial infection [17]. In mild COVID-19, PCT levels may remain within the normal range. However, there is an increase in PCT production during bacterial coinfection or in severe COVID-19, such as COVID-19 complicated with pneumonia or ARDS; this increase in PCT is enhanced by proinflammatory cytokines [18,19]. Elevations in D-dimer levels are also associated with an increased risk of mortality in patients with COVID-19 [20]. Thus, it may be biologically plausible that the weaning failure group, which contains a higher proportion of patients with ARDS requiring MV, would present with significantly higher baseline PCT, D-dimer, and TNF-α levels and these biomarkers were significantly associated with weaning failure in our study. Interestingly, baseline lymphocytes, LDH, and CRP, which are all independently associated with COVID-19 severity [21,22,23], did not predict weaning failure in our study. One possible explanation is that these biomarkers are not associated with the underlying inflammatory mechanisms related to severe acute respiratory syndrome coronavirus 2 (SARS-CoV-2) infection. For instance, a recent study demonstrated a heterogeneous CRP response among patients with COVID-19, with some patients with severe outcomes presenting with increased CRP levels and others presenting with increased neutrophil counts [24]. Initial chest radiography and viral load are associated with mortality and the need for ventilatory support [25,26], although neither of these factors predicted weaning failure in our study.

In contrast, NLR and endocan changes during the 7-day study period were useful in differentiating patients who failed to liberate from oxygen therapy at day 28 and the predictive value of a 7-day increase in endocan level was excellent. NLR, calculated as the ratio of neutrophil and lymphocyte counts, is an inflammatory biomarker that predicts mortality in critically ill patients [27]. In patients with COVID-19, a higher NLR appears to be an early risk factor for mortality [28]. In addition, serum endocan levels are elevated in several vascular pathologies, such as coronary artery disease and diabetes [29,30]. Previous studies have demonstrated that the SARS-CoV-2-induced hyperinflammatory response results in severe endothelial disruption and thrombus formation, which may cause pulmonary hypertension, increased dead space ventilation, and right heart failure [31]. Therefore, it may be biologically plausible that follow-up levels of endocan, a biomarker of endothelial injury, may be useful as a prognostic factor in patients with severe COVID-19 requiring oxygen therapy.

It is notable that four of the top nine biomarkers (Ang-2, Ang-2/Ang-1, and endocan [4-day and 7-day increases]) in the prediction model were biomarkers of endothelial injury. As mentioned above, the activation of inflammatory cascades and vasculitis are the central mechanisms underlying severe COVID-19. Thus, measures of endothelial injury may be useful in monitoring patients with COVID-19 not likely to wean from oxygen therapy. Angiotensin-converting enzyme 2 (ACE2) regulates the renin-angiotensin system by converting Ang-2 into Ang-(1–7) and Ang-1 into Ang-(1–9) [32]. SARS-CoV-2 binds to and downregulates ACE2, leading to over-accumulation of Ang-2 that in turn induces lung injury [33]. High levels of Ang-2 and Ang-2/Ang-1 and low levels of Ang-1 are expected in patients with severe COVID-19 due to the loss of ACE2 function. In the present study, biomarkers of epithelial injury did not predict weaning failure at the level of statistical significance. SP-D, a normal constituent surfactant produced by type II alveolar cells, has been independently associated with mortality and organ failure in patients with ARDS [34]. sRAGE level is also increased in lung epithelial injury and predicts the development of non-COVID-19 ARDS [35]. A recent study demonstrated that sRAGE was a good predictor of the need for MV as well as mortality among patients with COVID-19 [7], although sRAGE levels were not significantly higher among patients with mortality within 30 days of hospital admission when adjusting for disease severity. In addition, sRAGE levels did not differ between survivors and non-survivors with COVID-19 ARDS [36]. Therefore, sRAGE may predict severity and early mortality in severe COVID-19, although monitoring sRAGE for its associated complications, such as weaning failure, may not be useful.

The main strengths of the present study include prospectively collected blood samples and serial analyses of biomarkers. We evaluated dynamic changes in clinical variables and laboratory biomarkers in the weaning failure and success groups during the progression of COVID-19 with the aim of examining differences in the pathophysiology of severe COVID-19 and how these are associated with outcomes. Through longitudinal dissection of various parameters, we found that inflammatory changes (from baseline) may be better predictors of weaning failure than baseline parameters. This study also has some limitations. First, due to its retrospective single-center design, some potential selection bias and confounding variables were not considered. Second, the study cohort sample size and number of patients with weaning failure were relatively low, such that comprehensive statistical analyses (e.g., secondary and sensitivity analyses) could not be conducted and a comparison of different combinations of biomarkers with varying cut-off levels to predict weaning failure was not feasible in our study. Several potential biomarkers (baseline lymphocytes and IL-6) appeared to be associated with weaning failure, although they failed to reach statistical significance, possibly due to limited sample size. Third, due to the retrospective nature of the study, follow-up data were not available from each patient for several parameters, such as ferritin, PCT, D-dimer, and Ct value. Thus, the predictive values of these parameters during disease monitoring are unclear. Fourth, the high costs of some of the biomarkers evaluated in the current study may be a factor limiting widespread use in clinical practice.

## 5. Conclusions

In conclusion, abnormal levels of nine clinical and laboratory biomarkers provided moderate discrimination for the prediction of failure to liberate from oxygen therapy at day 28 following hospital admission in patients with severe COVID-19. Four of the nine biomarkers represent endothelial injury, which is a critical determinant of ARDS and organ failure, leading to decreased pulmonary function and prolonged oxygen support. These biomarkers may thus be useful indicators in the early rehabilitation of high-risk patients and may potentially be implemented in selecting patients for discharge to long-term care facilities or long-term in-home care (to optimize the hospital bed management). Our findings should be validated within highly powered studies. If confirmed, our findings will inform medical guidelines and medical decision-making.

## Figures and Tables

**Table 1:** Baseline and clinical characteristics of patients with severe COVID-19 according to oxygen therapy status at day 28 following hospital admission.

	Weaning Failure (*n* = 18)	Weaning Success (*n* = 38)	*p*
Age, years	76 (68–79)	68 (57–71)	0.01
Male sex	13 (72)	22 (58)	0.30
Body mass index, kg/m^2^	23.4 (21.0–30.8)	25.9 (24.6–28.0)	0.24
Comorbidities			
Diabetes	3 (17)	10 (26)	0.51
Hypertension	8 (44)	20 (53)	0.57
Chronic neurologic disease	2 (11)	3 (8)	0.65
Chronic lung disease	6 (33)	6 (16)	0.17
ARDS	9 (50)	8 (21)	0.03
Bacterial coinfection	5 (28)	5 (13)	0.26
qSOFA score	2 (1–2)	0 (0–1)	0.003
Initial oxygen therapy			0.001
Mechanical ventilation	13 (72)	9 (24)	
High-flow nasal cannula	5 (28)	17 (45)	
Supplemental oxygen	0	12 (32)	
Remdesivir	8 (44)	23 (61)	0.26
Steroid	18 (100)	38 (100)	
Vital signs and laboratory data			
Body temperature, °C	37.0 (36.4–38.1)	37.6 (36.9–38.0)	0.17
Mean blood pressure, mmHg	78 (68–97)	85 (73–97)	0.35
Heart rate, beats/min	93 (73–103)	86 (76–93)	0.26
Respiratory rate, breaths/min	25 (24–32)	20 (20–26)	0.006
SpO_2_/FiO_2_	157 (124–190)	208 (159–297)	0.004
Creatinine, mg/dL	0.8 (0.6–1.3)	0.7 (0.6–0.8)	0.09
White cell count, 1000/mm^3^	8.7 (6.2–13.2)	7.7 (5.5–10.1)	0.19
Lymphocytes, %	8.9 (4.3–13.3)	13.0 (7.1–19.0)	0.10
Neutrophil lymphocyte ratio	10.0 (6.2–23.5)	6.8 (4.1–14.0)	0.16
Platelet count, 1000/mm^3^	162 (134–219)	216 (179–248)	0.02
Total bilirubin, mg/dL	0.6 (0.4–0.7)	0.6 (0.5–0.8)	0.66
Lactate dehydrogenase, IU/L	436 (370–638)	404 (345–489)	0.16
C-reactive protein, mg/L	119 (56–214)	104 (62–148)	0.34
Ferritin, ng/mL ^1^	830 (427–1788)	862 (553–1260)	0.86
Procalcitonin, ng/mL ^2^	0.3 (0.1–0.6)	0.1 (0.1–0.2)	0.01
D-dimer, ug/mL ^3^	1.1 (0.8–6.8)	0.7 (0.5–1.0)	0.02
Glucose, mg/dL	170 (126–259)	153 (118–213)	0.55
Radiologic score	6 (3–7)	4 (3–6)	0.15
Cycle threshold value	21.8 (18.6–26.6)	26.1 (22.1–29.1)	0.06
Length of hospital stay, days	26 (12–40)	15 (12–22)	0.04
Hospital mortality	7 (39)	0	<0.001
Hospital-acquired infection	11 (61)	4 (11)	<0.001

Data are presented as medians (interquartile ranges) or numbers (%). ARDS: acute respiratory distress syndrome; COVID-19: coronavirus disease 2019; FiO_2_: fraction of inspired oxygen; SpO_2_: pulse oximetric saturation; qSOFA: quick Sequential Organ Failure Assessment. ^1^ Data available for 12 patients in the weaning failure group and 29 in the weaning success group. ^2^ Data available for 15 patients in the weaning failure group and 36 in the weaning success group. ^3^ Data available for 18 patients in the weaning failure group and 36 in the weaning success group.

**Table 2:** Plasma biomarkers of endothelial/epithelial injury and inflammation.

	Weaning Failure (*n* = 18)	Weaning Success (*n* = 38)	*p*
Ang-1, pg/mL	2199 (859–10,178)	6399 (2252–26,937)	0.08
Ang-2, pg/mL	1838 (1293–2813)	1050 (844–1587)	0.02
Ang-2/Ang-1	0.81 (0.27–2.34)	0.16 (0.04–0.42)	0.01
sTie2, pg/mL	11,343 (6894–15,052)	13,483 (10,425–17,899)	0.14
Ang-1/sTie2	0.28 (0.09–1.13)	0.56 (0.16–1.57)	0.14
Endocan, pg/mL	1026 (421–1509)	877 (401–1577)	0.96
ICAM-1, pg/mL	289,403 (191,651–506,711)	347,428 (208,226–459,056)	0.99
IL-6, pg/mL	37.5 (11.2–58.9)	17.4 (4.6–41.7)	0.08
sRAGE, pg/mL	6433 (2866–13,087)	4904 (2253–6575)	0.14
SP-D, pg/mL	15,618 (2842–28,038)	6385 (1709–12,215)	0.09
Syndecan-1, pg/mL	9000 (5581–12,353)	5969 (4734–7670)	0.06
TNF-α, pg/mL	7.8 (6.5–11.6)	5.7 (4.1–7.9)	0.006
vWF, pg/mL	3158 (1305–5910)	2613 (1204–4939)	0.99

Data are presented as medians (interquartile ranges). Ang-1: angiopoietin-1; Ang-2: angiopoietin-2; ICAM-1: intercellular adhesion molecule-1; IL-6: interleukin-6; sTie2: soluble form of the Tie2 receptor; sRAGE: soluble receptor for advanced glycation end-products; SP-D: surfactant protein D; TNF-α: tumor necrosis factor-α; vWF: von Willebrand factor.

**Table 3:** Models for predicting failure of liberation from oxygen therapy at day 28 following hospital admission through the top nine performing biomarkers.

	AUC (95% CI)	*p*
qSOFA score	0.73 (0.59–0.87)	0.005
SpO_2_/FiO_2_	0.74 (0.60–0.88)	0.004
Procalcitonin	0.73 (0.57–0.88)	0.01
D-dimer	0.70 (0.54–0.85)	0.02
Ang-2	0.70 (0.54–0.86)	0.02
Ang-2/Ang-1	0.71 (0.56–0.86)	0.01
Endocan (day 4–baseline)	0.75 (0.60–0.90)	0.003
Endocan (day 7–baseline)	0.81 (0.67–0.94)	<0.001
TNF-α	0.73 (0.60–0.87)	0.006

Ang-1: angiopoietin-1; Ang-2: angiopoietin-2; AUC: area under the curve; CI: confidence interval; FiO_2_: fraction of inspired oxygen; SpO_2_: pulse oximetric saturation; qSOFA: quick Sequential Organ Failure Assessment; TNF-α: tumor necrosis factor-α.

## Data Availability

The data presented in this study are available within the article or Supplementary Material.

## Supplementary Materials

The following are available online at https://www.mdpi.com/article/10.3390/jpm11100974/s1, Table S1: Clinical variables and laboratory parameters during hospitalization according to oxygen therapy status at day 28, Table S2: Models for predicting failure of liberation from oxygen therapy at day 28 following hospital admission through clinical variables and laboratory parameters.
