# Supplementary material for: Biomarkers Associated with Failure of Liberation from Oxygen Therapy in Severe COVID-19: A Pilot Study
Source: J Pers Med. 2021 Sep 29;11(10):974. doi: 10.3390/jpm11100974 (PMC8538650; doi:10.3390/jpm11100974)
Supplement: Supplementary file 1 [file jpm-11-00974-s001.zip › jpm-1402193-supplementary.pdf]

Table S1. Clinical variables and laboratory parameters during hospitalization according to oxygen therapy status at day 28.

	Baseline	Day 4	Day 7	AUC	*p*
SpO_2/FiO_2					0.87
Weaning failure	157 (124–190)	204 (188–238)	192 (176–214)	38 (15–62)	
Weaning success	208 (159–297)	241 (220–317)	292 (248–350)	32 (-4 to 69)	
Radiologic score					0.95
Weaning failure	6 (3–7)	4 (2–6)	2 (2–4)	-1 (-2 to 0)	
Weaning success	4 (3–6)	3 (2–4)	2 (2–3)	-1 (-2 to 0)	
Neutrophil lymphocyte ratio					0.04
Weaning failure	10.0 (6.2–23.5)	12.3 (9.6–23.0)	16.6 (8.8–23.0)	1.8 (0.5–6.2)	
Weaning success	6.8 (4.1–14.0)	6.7 (4.1–10.5)	6.8 (4.3–9.6)	-0.4 (-5.1–2.1)	
Lactate dehydrogenase, IU/L					0.26
Weaning failure	436 (370–638)	403 (380–504)	424 (336–476)	-24 (-93 to 11)	
Weaning success	404 (345–489)	332 (293–392)	282 (251–359)	-63 (-104 to -7)	
C-reactive protein, mg/L					0.61
Weaning failure	119 (56–214)	40 (29–101)	23 (14–83)	-62 (-118 to -24)	
Weaning success	104 (62–148)	28 (18–43)	7 (2–25)	-53 (-88 to -27)	
Ang-1, pg/mL					0.96
Weaning failure	2199 (859–10178)	3690 (2070–5623)	3381 (1980–5962)	1056 (-1036 to 2430)	
Weaning success	6399 (2252–26937)	5728 (2807–18794)	6572 (3993–16234)	822 (-8132 to 3321)	
Ang-2, pg/mL					0.19
Weaning failure	1838 (1293–2813)	1003 (675–1856)	1394 (908–1835)	-516 (-1207 to -64)	
Weaning success	1050 (844–1587)	729 (583–1103)	816 (544–1259)	-172 (-555 to -126)	
Ang-2/Ang-1					0.37
Weaning failure	0.81 (0.27–2.34)	0.18 (0.11–0.81)	0.34 (0.14–0.62)	-0.07 (-1.59 to 0.03)	
Weaning success	0.16 (0.04–0.42)	0.13 (0.05–0.26)	0.11 (0.07–0.18)	-0.03 (-0.21 to 0.03)	
sTie2, pg/mL					0.29
Weaning failure	11343 (6894–15052)	10408 (6370–13960)	13187 (8817–15993)	169 (-4463 to 2276)	
Weaning success	13483 (10425–17899)	11551 (9076–14293)	12039 (9597–16091)	-1395 (-3563 to 418)	
Ang-1/sTie2					0.69
Weaning failure	0.28 (0.09–1.13)	0.44 (0.20–0.70)	0.27 (0.22–0.72)	0.08 (-0.18 to 0.28)	
Weaning success	0.56 (0.16–1.57)	0.62 (0.24–1.71)	0.68 (0.29–0.97)	0.13 (-0.54 to 0.61)	

Endocan, pg/mL					0.001
Weaning failure	1026 (421–1509)	1249 (802–3182)	1964 (957–3133)	688 (235–1257)	
Weaning success	877 (401–1577)	713 (427–1333)	666 (383–1325)	-59 (-318 to 174)	
ICAM-1, pg/mL					0.83
Weaning failure	289403 (191651–506711)	289806(155966–423281)	270908 (171758–424315)	-21685 (-78258 to -3739)	
Weaning success	347428 (208226–459056)	310264 (163131–423758)	309873 (174163–408408)	-24394 (-51910 to -4779)	
IL-6, pg/mL					0.65
Weaning failure	37.5 (11.2–58.9)	11.4 (7.4–18.4)	9.2 (4.6–36.3)	-21.3 (-36.0 to 0.9)	
Weaning success	17.4 (4.6–41.7)	4.3 (2.8–6.5)	2.8 (1.5–7.0)	-10.4 (-25.7 to -0.6)	
sRAGE, pg/mL					0.53
Weaning failure	6433 (2866–13087)	2815 (1455–5566)	1988 (960–4043)	-2164 (-7385 to -449)	
Weaning success	4904 (2253–6575)	2228 (1455–3263)	1217 (820–1766)	-1725 (-3057 to -593)	
SP-D, pg/mL					0.007
Weaning failure	6385 (1709–12215)	9986 (3763–21967)	5497 (1851–11808)	-450 (-9460 to 3218)	
Weaning success	7116 (2524–14272)	13460 (8405–20337)	11079 (7592–19257)	3731 (856–6590)	
Syndecan-1, pg/mL					0.37
Weaning failure	9000 (5581–12353)	10266 (5824–14762)	11333 (8237–16026)	1213 (-528 to 4045)	
Weaning success	5969 (4734–7670)	7626 (5693–9690)	7509 (5973–10727)	863 (-165 to 2117)	
TNF-α, pg/mL					0.85
Weaning failure	7.8 (6.5–11.6)	6.3 (5.2–9.0)	6.4 (5.7–8.7)	-0.7 (-2.3 to 0.7)	
Weaning success	5.7 (4.1–7.9)	4.5 (3.3–6.1)	4.4 (3.1–5.8)	-0.7 (-1.6 to 0.3)	
vWF, pg/mL					0.67
Weaning failure	3158 (1305–5910)	1968 (1208–3297)	1977 (1377–3265)	-354 (-1601 to 304)	
Weaning success	2613 (1204–4939)	2096 (1044–3818)	2324 (1050–3648)	-123 (-612 to 261)	

The median (interquartile range) values for the AUCs were calculated for variables with repeated measurements (baseline, day 4, and day 7). Ang-1: angiopoietin-1; Ang-2: angiopoietin-2; AUC: area under the curve; FiO_2: fraction of inspired oxygen; ICAM-1: intercellular adhesion molecule-1; IL-6: interleukin-6; SpO_2: pulse oximetric saturation; sTie2: soluble form of the Tie2 receptor; sRAGE: soluble receptor for advanced glycation end-products; SP-D: surfactant protein D; TNF-α: tumor necrosis factor-α; vWF: von Willebrand factor.

Table S2. Models for predicting failure of liberation from oxygen therapy at day 28 following hospital admission through clinical variables and laboratory parameters.

	**AUC (95% CI)**	***p***
qSOFA score	**0.73 (0.59–0.87)**	0.005
SpO_2/FiO_2	**0.74 (0.60–0.88)**	0.004
Day 4 – baseline	0.42 (0.26–0.58)	0.34
Day 7 – baseline	0.64 (0.49–0.79)	0.10
Radiologic score	0.62 (0.45–0.79)	0.15
Day 4 – baseline	0.54 (0.36–0.71)	0.67
Day 7 – baseline	0.45 (0.27–0.63)	0.57
Cycle threshold value	0.66 (0.50–0.82)	0.06
Lymphocytes	0.64 (0.48–0.80)	0.10
Day 4 – baseline	0.63 (0.49–0.78)	0.11
Day 7 – baseline	0.64 (0.50–0.78)	0.10
Neutrophil lymphocyte ratio	0.62 (0.46–0.78)	0.16
Day 4 – baseline	0.69 (0.54–0.84)	0.03
Day 7 – baseline	0.65 (0.49–0.82)	0.07
Lactate dehydrogenase	0.62 (0.46–0.78)	0.16
Day 4 – baseline	0.58 (0.42–0.74)	0.34
Day 7 – baseline	0.65 (0.49–0.80)	0.08
C-reactive protein	0.58 (0.41–0.75)	0.34
Day 4 – baseline	0.46 (0.29–0.64)	0.64
Day 7 – baseline	0.50 (0.31–0.68)	0.96
Procalcitonin	**0.73 (0.57–0.88)**	0.01
D-dimer	**0.70 (0.54–0.85)**	0.02
Ang-1	0.65 (0.49–0.81)	0.08
Day 4 – baseline	0.51 (0.35–0.66)	0.93
Day 7 – baseline	0.48 (0.33–0.63)	0.83
Ang-2	**0.70 (0.54–0.86)**	0.02
Day 4 – baseline	0.38 (0.19–0.56)	0.15
Day 7 – baseline	0.35 (0.19–0.52)	0.08
Ang-2/Ang-1	**0.71 (0.56–0.86)**	0.01
Day 4 – baseline	0.46 (0.27–0.65)	0.62
Day 7 – baseline	0.42 (0.23–0.60)	0.31
sTie2	0.38 (0.21–0.54)	0.14
Day 4 – baseline	0.57 (0.39–0.76)	0.40
Day 7 – baseline	0.62 (0.43–0.80)	0.17
Ang-1/sTie2	0.62 (0.46–0.78)	0.14
Day 4 – baseline	0.52 (0.37–0.67)	0.82
Day 7 – baseline	0.54 (0.38–0.69)	0.67
Endocan	0.50 (0.33–0.66)	0.96
Day 4 – baseline	**0.75 (0.60–0.90)**	0.003
Day 7 – baseline	**0.81 (0.67–0.94)**	<0.001
ICAM-1	0.50 (0.33–0.67)	0.99
Day 4 – baseline	0.54 (0.36–0.72)	0.62
Day 7 – baseline	0.50 (0.33–0.67)	0.99
IL-6	0.64 (0.49–0.80)	0.08
Day 4 – baseline	0.44 (0.26–0.61)	0.46
Day 7 – baseline	0.48 (0.29–0.67)	0.81
sRAGE	0.62 (0.44–0.80)	0.14

Day 4 – baseline	0.41 (0.23–0.59)	0.27
Day 7 – baseline	0.47 (0.28–0.66)	0.71
SP-D	0.64 (0.48–0.80)	0.09
Day 4 – baseline	0.25 (0.10–0.40)	0.003
Day 7 – baseline	0.30 (0.14–0.46)	0.02
Syndecan-1	0.66 (0.49–0.83)	0.06
Day 4 – baseline	0.55 (0.36–0.73)	0.59
Day 7 – baseline	0.64 (0.49–0.79)	0.09
TNF-α	**0.73 (0.60–0.87)**	0.006
Day 4 – baseline	0.50 (0.33–0.68)	0.96
Day 7 – baseline	0.46 (0.28–0.64)	0.62
vWF	0.50 (0.33–0.67)	0.99
Day 4 – baseline	0.44 (0.28–0.60)	0.49
Day 7 – baseline	0.51 (0.35–0.68)	0.88

Ang-1: angiopoietin-1; Ang-2: angiopoietin-2; AUC: area under the curve; CI: confidence interval; FiO_2: fraction of inspired oxygen; ICAM-1: intercellular adhesion molecule-1; IL-6: interleukin-6; SpO_2: pulse oximetric saturation; qSOFA: quick Sequential Organ Failure Assessment; sTie2: soluble form of the Tie2 receptor; sRAGE: soluble receptor for advanced glycation end-products; SP-D: surfactant protein D; TNF-α: tumor necrosis factor-α; vWF: von Willebrand factor.
